# Efficacy of prophylactic selective arterial embolization for renal angiomyolipomas: identifying predictors of 50% volume reduction

**DOI:** 10.1186/s42155-020-00179-2

**Published:** 2020-11-21

**Authors:** Hidenari Hongyo, Hiroki Higashihara, Keigo Osuga, Eiji Kashiwagi, Shinya Kosai, Keisuke Nagai, Kaishu Tanaka, Yusuke Ono, Takeshi Ujike, Motohide Uemura, Ryoichi Imamura, Norio Nonomura, Noriyuki Tomiyama

**Affiliations:** 1 Department of Diagnostic and Interventional Radiology, Osaka University Graduate School of Medicine, 2-2 Yamadaoka, Suita, Osaka, Japan; 2 Department of Diagnostic Radiology, Osaka Medical College, 2-7 Daigaku-machi, Takatsuki, Osaka, Japan; 3 Department of Urology, Osaka University Graduate School of Medicine, 2-2 Yamadaoka, Suita, Osaka, Japan

**Keywords:** Kidney neoplasms, Angiomyolipoma, Embolization, Therapeutic, Tuberous sclerosis

## Abstract

**Background:**

Transcatheter arterial embolization (TAE) has been widely performed for renal angiomyolipomas (AMLs) as prophylaxis or emergency treatment. On the other hand, mammalian target of rapamycin (mTOR) inhibitors have recently been used for tuberous sclerosis (TSC)-related AMLs, and no comparison between the effectiveness of mTOR inhibitors versus prophylactic selective TAE has yet been performed.

Therefore, the purpose of this study was to evaluate the efficacy of TAE for AML tumor volume reduction and predictors of tumor volume decrease over 50%, with reference to the EXIST-2 trial.

**Methods:**

A total of 44 patients who underwent 48 prophylactic embolization procedures for 50 AMLs in a single institution between 2004 and 2018 were included. Indications for TAE of AMLs were tumor size ≥4 cm or aneurysm ≥5 mm in diameter on contrast-enhanced computed tomography (CECT). Microspheres, ethanol, and micro-coils were used as embolic agents. The percentage volume reduction from before TAE to the minimum volume during follow-up after TAE was calculated, and predictors for 50% volume reduction were identified by univariate and multivariate binary logistic regression analyses.

**Results:**

The technical success rate was 100% (50 of 50). No severe acute complications related to the procedure were encountered. Tumor volume reduction of ≥50% was observed in 35/50 AMLs. There was a significant difference in the rate of tumor volume reduction of 50% between the presence and absence of an aneurysm ≥5 mm and between tumor diameter ≥ 70 mm and < 70 mm on univariate analysis. On multivariate analysis, tumor diameter < 70 mm was the only independent predictor of significant tumor volume reduction after TAE.

**Conclusion:**

Prophylactic selective TAE for AMLs has good tumor-reduction effects, especially for AMLs with tumor diameter < 70 mm.

## Background

Renal angiomyolipoma (AML) is an uncommon benign tumor, representing 1–3% of all renal tumors (Fujii et al. 1995; Steiner et al. 1993; Nelson and Sanda 2002; Harabayashi et al. 2004). AMLs are classified broadly into two types: the sporadic type, constituting 80% of renal AMLs; and AMLs associated with tuberous sclerosis (TSC), constituting 20% of all renal AMLs (Fujii et al. 1995; Steiner et al. 1993; Harabayashi et al. 2004). AMLs are identifiable on computed tomography (CT) and magnetic resonance imaging (MRI) due to their fat content (Flum et al. 2016). In symptomatic cases, the most common symptom is flank pain, followed by a palpable mass and hematuria (Nelson and Sanda 2002). AMLs are also considered hypervascular tumors, with fine feeding arteries accompanied by microaneurysms. These tumors are thus at risk of intratumoral hemorrhage, rupture, and massive retroperitoneal or intraperitoneal hemorrhage, which can prove life-threatening (Nelson and Sanda 2002; Harabayashi et al. 2004; Soulen et al. 1994). Radical and partial nephrectomy, transcatheter arterial embolization (TAE), and ablative therapies, including cryoablation and radiofrequency ablation, are considered treatment options (Flum et al. 2016). Of them, TAE is widely performed for AMLs as the first choice for prophylaxis or emergency treatment for bleeding (Steiner et al. 1993; Van Baal et al. 1994; Kennelly et al. 1994). Previous reports have generally suggested that prophylactic treatments such as TAE should be performed for asymptomatic AMLs ≥4 cm in diameter or AMLs with microaneurysms ≥5 mm in the feeding artery (Yamakado et al. 2002). Needless to say, symptomatic tumors, such as those with hemorrhage, should always be treated. On the other hand, mammalian target of rapamycin (mTOR) inhibitors have recently come to be widely used for TSC-AMLs. According to the International Tuberous Sclerosis Complex Consensus Conference 2012, mTOR inhibitors have been recommended as a first-line treatment to reduce the size of renal AMLs associated with TSC (Northrup et al. 2013). On the other hand, proper selection and use of drugs such as mTOR inhibitors or prophylactic selective TAE have not yet been studied. Therefore, the purpose of this study was to evaluate the efficacy of TAE for tumor volume reduction of AMLs and identify predictors of tumor volume decrease over 50%, with reference to the EXIST-2 trial (Bissler et al. 2013).

## Material and methods

### Study design and patient population

This was a single-center, retrospective study. Our institutional review board approved the study. The need to obtain informed consent was waived due to the retrospective nature of the study design.

Between August 2004 and March 2018, a total of 48 consecutive patients (sporadic-AML (s-AML) *n* = 19; TSC-AML *n* = 29) who had undergone TAE were enrolled. Indications for TAE were tumor diameter ≥ 4 cm, a feeding artery accompanied with a microaneurysm ≥5 mm in diameter on dynamic contrast-enhanced computed tomography (CECT), or symptoms such as retroperitoneal bleeding due to tumor rupture or hematuria. In our institution, the treatment strategy for all patients with renal AML was decided by interventional radiologists and urologists in a consensus conference. Five patients underwent emergency TAE for tumor rupture using gelatin sponges, and 2 patients underwent TAE using only absolute ethanol as the embolic agent. These patients were excluded because the embolization methods differed from the standard method of prophylactic TAE at our institute. Two patients with no imaging follow-up were also excluded. Thus, a total of 44 patients with 50 AMLs were evaluated. The patients included 12 males and 32 females, with a mean age of 34.5 years (range, 15–70 years). There were 3 cases with symptoms of flank pain and 3 cases with symptoms of hematuria. Blood supply from other than renal arteries included 6 renal capsular arteries, 1 adrenal artery, and 1 lumbar artery. The patients’ characteristics are shown in Table 1, and the AMLs’ characteristics are shown in Table 2.

**Table 1:** Patients’ background characteristics

Variable	Volume reduction ≥50% *n* = 30	Volume reduction < 50% *n* = 14	*P*-value
Sex, n (%)			
Male	9 (30)	3 (21.4)	
Female	21 (70)	11 (78.6)	0.55
Age (mean y ± SD)	36.2 (±15.7)	30.5 (±12.9)	0.23
Location, n (%)			
Unilateral	16 (53.3)	3 (21.4)	
Bilateral	14 (46.7)	11 (78.6)	0.047
Masses, n (%)			
Single	14 (46.7)	3 (21.4)	
Multiple	16 (53.3)	11 (78.6)	0.11
Etiology, n (%)			
Sporadic	13 (43.3)	2 (14.3)	
^a^TSC-related	17 (56.7)	12 (85.7)	0.058
Symptoms, n (%)			
Symptomatic	4 (13.3)	2 (14.3)	
Asymptomatic	26 (86.7)	12 (85.7)	0.93

^a^*TSC* tuberous sclerosis

**Table 2:** AMLs’ characteristics

Variables	Volume reduction ≥50% *n* = 35	Volume reduction < 50% *n* = 15	*P*-value
Tumor diameter (mean mm ± SD)	65.9 ± 23.4	80.0 ± 26.3	0.00
Aneurysm < 5 mm or none, n (%)	25 (71.4)	6 (40.0)	0.036
Embolic agent, n (%)			
^a^SAP-MS	12 (34.3)	3 (20.0)	
Embozene, Embosphere	23 (65.7)	12 (80.0)	0.31
Image evaluation time after TAE (mean months±SD)	31.6 ± 26.0	27.3 ± 29.0	0.61

^a^*SAP-MS* sodium acrylate and vinyl alcohol copolymer

### TAE procedure for AMLs

**Fig. 1:**
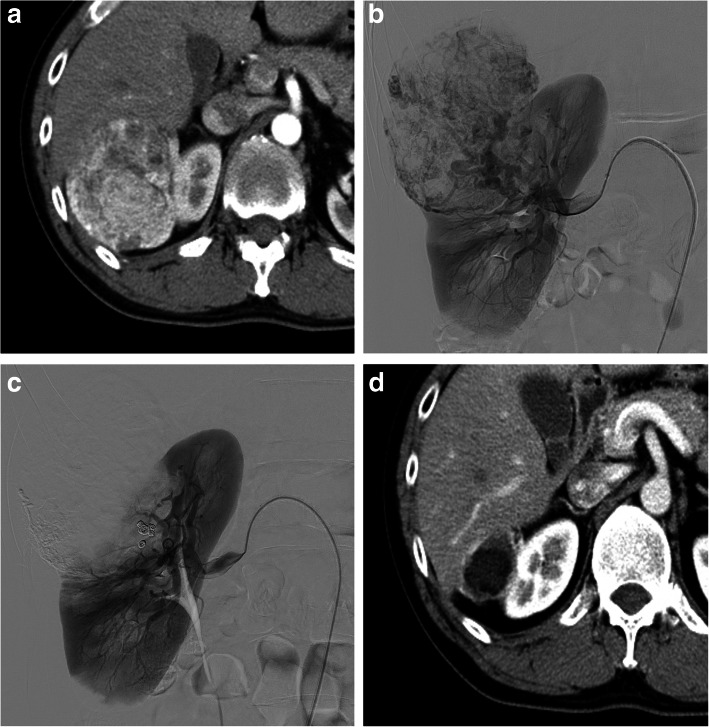
A 35-year-old man with a sporadic AML. Contrast-enhanced CT shows an exophytic, well-enhanced, right renal AML, 64 mm in diameter (**a**). Right renal angiography before embolization shows tumor staining and tortuous feeding arteries with a microaneurysm (**b**). Tumor stain and feeding arteries disappear after embolization using microspheres, absolute ethanol, and microcoils (**c**). Contrast-enhanced CT 2 years after TAE shows marked shrinkage of the embolized tumor with necrotic changes (**d**)

**Fig. 2:**
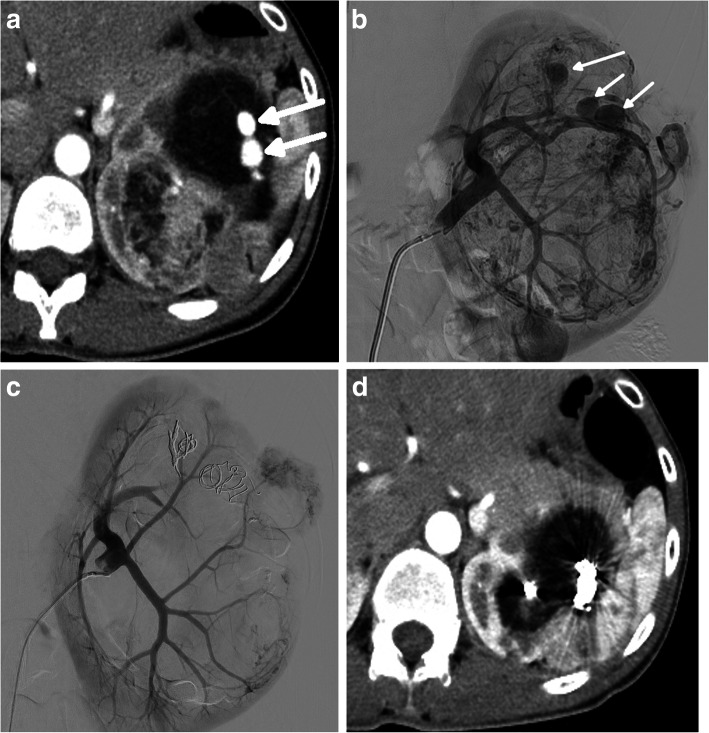
A 34-year-old woman with a TSC-AML. Contrast-enhanced CT shows multiple left renal AMLs. The tumor in the ventral lateral position in the left kidney contains a microaneurysm, 11 mm in diameter (**a**) (white arrows). Left renal angiography before embolization shows tumor stain and several microaneurysms (white arrows). Normal left renal parenchyma is compressed by the embolized tumor (**b**). Embolized tumor stain and microaneurysms disappear after embolization using microspheres, absolute ethanol, and microcoils. Normal renal parenchyma is retained, and a residual untreated tumor is shown (white arrows) (**c**). Contrast-enhanced CT 2 years after TAE shows a reduction in tumor volume and no residual aneurysm (**d**)

During the study period, eight interventional radiologists were involved in this treatment, and the choice of embolic materials or embolization technique largely depended on each operator. The routine procedures were performed as follows. A 4-Fr sheath (Supersheath; Medikit Co., Tokyo, Japan) or 4.5-Fr guiding sheath (45 Parent Plus; Hanaco Medical Co., Saitama, Japan) was inserted via right trans-femoral access under local anesthesia. Angiography at the renal artery trunk was performed in all cases to evaluate tumor location and feeding arteries (Figs. 1b, 2b). In complicated cases, 3-dimensional digital subtraction angiography (DSA) was also performed. After diagnostic angiography, a microcatheter (Masters Parkway Soft; Asahi Intecc Co., Nagoya, Japan) was selectively advanced into the feeding artery to avoid embolization of normal renal parenchyma. The method for TAE of AMLs was as follows. First, spherical microspheres were gently injected into the feeding artery until tumor stain disappeared but blood flow of the feeding artery was preserved. Three types of microspheres were used. From 2004 to 2014, SAP-MS (sodium acrylate and vinyl alcohol copolymer) and Embozene (CeloNova Biosciences, Newnan, GA, USA) were used, and from 2014, Embosphere (Merit Medical, Rockland, MA, USA) was used as the embolic material. Absolute ethanol was additionally injected to ablate the endothelium of the feeding artery. Approximately 1 ml of ethanol was injected repeatedly until blood flow became static. Microcoils were deployed to the microaneurysm or the proximal feeding artery in cases with blood flow remaining in the feeding artery (Figs. 1c, 2c). The types of coils were selected according to the judgment of the operator, and they included pushable coils (Tornado, Hilal, and Nester coils; Cook, Bloomington, IN, USA; C-STOPPER coil, Piolax Medical Devices, Yokohama, Japan) and detachable microcoils (Penumbra SMART COIL; Penumbra, Alameda, CA, USA). The feeding artery was embolized while preserving as much normal renal parenchyma as possible. The endpoint of embolization was defined as the disappearance of or marked decrease in tumor stain (Figs. 1c, 2c).

### Definitions of technical success and complications

Technical success was defined as achievement of disappearance of tumor stain and of micro-aneurysms with stasis of the feeding artery. Complications related to TAE were classified according to the criteria of the Society of Interventional Radiology as major or minor (Khalilzadeh et al. 2017).

### Measurement of change in embolized tumor size

Contrast-enhanced CT was used to measure changes in embolized tumor volume. Tumor volume before TAE and the minimum tumor volume during follow-up after TAE were measured, and the percentage reduction in tumor volume was calculated (Figs. 1a, d, 2a, d). All measurements were performed by 2 radiologists (H. Hi. and H. Ho. with 18 years and 8 years of experience, respectively).

Tumor area was obtained by manually drawing the tumor margin on each CT image. Tumor volume was obtained by multiplying the tumor area in each slice by the slice thickness (1–5 mm on CT). Follow-up observation was terminated when tumor re-growth was confirmed on contrast-enhanced CT. In the TSC-AML group, follow-up was also terminated after administration of an mTOR inhibitor was started. Volume reduction rates of tumors during follow-up were calculated. A tumor volume reduction of ≥50% after TAE was defined as a significant decrease, according to the EXIST-2 trial (Bissler et al. 2013).

### Statistical analysis

Continuous variables with normal distributions are reported as means ± standard deviation (SD), whereas non-normally distributed data are reported as medians and interquartile range. Differences between two groups were evaluated by the Chi-squared test for categorical measures (sex, symptoms, tumor location, number of tumors, presence of aneurysm ≥5 mm, embolic agent, etiology). Normally and non-normally distributed continuous variables (age, image evaluation time, tumor diameter) were analyzed using Student’s *t*-test and Welch’s test, respectively. Univariate and multivariate binary logistic regression analyses were performed to identify significant predictors of 50% tumor volume reduction. A *P* value < 0.05 was considered significant (2-sided). SPSS version 24 was used for the analysis.

## Results

A total of 44 patients underwent 48 prophylactic embolization procedures for 50 AMLs. The patients’ mean age was 34.5 ± 15.1 years, mean image evaluation time was 30.28 ± 27.0 months, and mean follow-up time was 49.9 ± 36.3 months. The technical success rate was 100% (48 of 48). No severe acute complications related to the procedure were encountered. The mean tumor volume reduction rate of all AMLs was 66.0% ± 24.4%. The mean tumor volume reduction rate of s-AMLs was 72.9% ± 21.5%, and that of TSC-AMLs was 63.5% ± 25.2%.

Tumor volume reduction of ≥50% was observed in 35/50 AMLs (TSC 22/35, sporadic 13/15). Table 1 also shows the comparison between the group with tumor volume reduction of ≥50% and the group with tumor volume reduction of < 50%. Of the variables shown in Table 1 and Table 2, presence of aneurysm (≥5 mm) and tumor diameter before TAE were significantly (*P* < 0.05) different between the two groups. There were no significant differences in age, sex, absence of symptoms, number of masses, location, image evaluation time, and embolic agent between the two groups. The results of the univariate and multivariate binary logistic regression analyses are shown in Table 3. Of the variables included in the univariate analysis, presence of aneurysm ≥5 mm and tumor diameter (COR = 0.267, 95%CI 0.075–0.947; COR = 0.190, 95%CI 0.050–0.725) were significantly associated with significant tumor volume reduction after TAE. On multivariate analysis, tumor diameter ≥ 70 mm (COR = 0.190, 95%CI 0.050–0.725) was the only independent predictor of significant tumor volume reduction after TAE.

**Table 3:** Univariate and multivariate analyses

Variables	Univariate		Multivariate	
	odds ratio (95%CI)	*P*-value	odds ratio (95%CI)	*P*-value
Sex				
Male vs. Female	0.636 (0.142–2.842)	0.293	1.090 (0.182–6.527)	0.925
Age				
≥ 30 vs. < 30	1.934 (0.569–6.580)	0.29		
≥ 35 vs. < 35	1.588 (0.465–5.419)	0.46		
≥ 40 vs. < 40	3.841 (0.746–19.781)	0.09	3.276 (0.592–18.130)	0.174
Etiology				
Sporadic vs. ^b^TSC	3.841 (0.746–19.781)	0.09	0.150 (0.005–4.774)	0.283
Unilateral vs. Bilateral	4.190 (0.969–18.122)	0.086	0.529 (0.103–2.710)	0.445
Single vs. multiple	0.312 (0.072–1.348)	0.11	^a^NA	^a^NA
Aneurysm ≥5 mm vs. < 5 mm or none	0.267 (0.075–0.947)	0.03	0.478 (0.101–2.256)	0.351
Tumor diameter mm				
≥ 65 vs. < 65	0.242 (0.064–0.916)	0.02		
≥ 70 vs. < 70	0.190 (0.050–0.725)	0.011	0.190 (0.050–0.725)	0.015
≥ 75 vs. < 75	0.261 (0.072–0.939)	0.03		.
^c^SAP vs. ^d^EZ, ^e^ES	0.479 (0.113–2.032)	0.31	0.412 (0.083–2.051)	0.279

^a^*NA* Not available, ^b^*TSC* tuberous sclerosis, ^c^*SAP-MS* sodium acrylate and vinyl alcohol copolymer, ^d^*EZ* Embozene, ^e^*ES* Embosphere

Treatment outcomes of TAE for s-AMLs and TSC-AMLs are shown in Table 4. One patient with an s-AML showed tumor bleeding after TAE due to incidental trauma. There were no other cases of bleeding during the follow-up period after TAE. None of the symptomatic cases showed relapse of symptoms after TAE. The regrowth rate after TAE tended to be higher for TSC-AMLs (31.4%, 11/35) than for s-AMLs (20%, 3/15), but the difference was not significant (*p* = 0.41). Mean regrowth time was 38.5 (±28.0) months. Of the s-AML patients, one underwent partial nephrectomy, nephrectomy was planned for one patient, and another patient remained under observation. Of the TSC-AML patients, seven patients had started everolimus, an m-TOR inhibitor. The other 4 patients remained under observation.

**Table 4:** Outcomes of TAE for sporadic and tuberous sclerosis-related AMLs

	Sporadic	^a^TSC	ALL	*P*-value
^b^AMLs, n	15	35	50	
Mean volume reduction rate, % (±SD)	72.9 (±21.5)	63.5 (±25.2)	66.0 (±24.4)	0.27
Regrowth, n (%)	3 (20.0%)	11 (31.4%)	14 (28.0%)	0.41
Aneurysm ≥5 mm before TAE, n (%)	2 (13.3%)	17 (48.6%)	19 (38%)	0.019
Aneurysm ≥5 mm after TAE, n (%)	0 (0%)	1 (2.9%)	1 (2.0%)	

^a^*TSC* tuberous sclerosis, ^b^*AML* angiomyolipomas

Intratumoral aneurysms > 5 mm before TAE were significantly more common in TSC-AML patients (48.6%, 17/35) than in s-AML patients (13.3%, 2/15) (*p* = 0.019). After TAE, there was only one residual aneurysm > 5 mm left.

## Discussion

The results of the present study showed that the tumor volume of AMLs decreased significantly after TAE. On multivariate analysis, tumor diameter < 70 mm before TAE was an independent predictor of a significant AML volume reduction after TAE.

Recently, m-TOR inhibitors have been used for TSC-related disease. These m-TOR inhibitors can reportedly cause safe tumor shrinkage, and they are now considered the first-line therapy for growing AMLs > 3 cm in diameter. On the other hand, TAE is the second-line therapy according to the International Tuberous Sclerosis Complex Consensus Conference 2012 (Northrup et al. 2013).

Tumor volume reduction over 50% was defined as a significant treatment effect in the present study to compare with that of mTOR inhibitors for AMLs in the EXIST-2 trial. In the present study, TAE was effective for reducing tumor volume of AMLs, especially AMLs less than 7 cm in diameter. Tumor volume reduction of 50% or more was seen in 70% of all AMLs and 63% of TSC-AMLs. These therapeutic results surpass those of everolimus in the EXIST-2 trial (42%). Based on the present study and a previous report, for AMLs with diameters of 4 to 7 cm, TAE can be considered effective because of its tumor volume reduction effect (Yamakado et al. 2002).

There was no significant correlation between AML etiology and tumor volume reduction in any analysis in the present study. Villalta et al. reported that there was no difference in tumor size reduction in patients with and without TSC, consistent with the present study (Villalta et al. 2011). In contrast, Lin et al. reported that TSC background was an independent predictor of a significant reduction in tumor size (Lin et al. 2018). One of the reasons may be due to the difference in embolic agents. Villalta et al. used microparticles, whereas Lin et al. used lipiodol/bleomycin emulsion with polyvinyl alcohol (PVA) as the embolic agent. Another reason may be the difference in criteria for effective tumor reduction. A prospective study is needed to clarify the difference in the efficacy of TAE between s-AMLs and TSC-AMLs.

The present study also showed that the presence of aneurysms larger than 5 mm was not correlated with the tumor-reducing effect. On the other hand, the therapeutic effect on aneurysms was satisfactory (18/19, 94.7%).

AMLs do not disappear with the use of mTOR inhibitors, since tumor regrowth is observed with withdrawal, and the effects on aneurysm reduction have yet to be clarified (Bissler et al. 2013; Bissler et al. 2016). Even if a patient with a TSC-AML is considered appropriate for treatment with mTOR inhibitors, TAE should be considered first if aneurysms ≥5 mm in diameter are observed. The present results suggest that TAE for TSC-AMLs is effective for embolizing aneurysms, and TAE thus seems to be a useful choice for preventing tumor bleeding and rupture.

Several limitations should be considered when interpreting the results of this study. In this study, the treatment effects of TAE in our institute were compared with those of an mTOR inhibitor in the EXIST-2 trial. A prospective study comparing the effectiveness of TAE and an mTOR inhibitor for AML is needed in the future. Generally, TAE procedures for AML have not been standardized. The procedural method also depended on individual operators in this study. However, the operators shared a broad consensus view about TAE procedures for AML in our institute, so the present results were considered to have been obtained under relatively standardized conditions. Further studies to examine the effects of TAE for AML based on clearly standardized procedures are needed.

## Conclusion

Based on the present results, TAE offers good tumor-reduction effects for AMLs, especially for AMLs less than 7 cm in diameter.

## Data Availability

All data gathered or analyzed in this study are included in this article.

## Abbreviations

AML: Angiomyolipoma
CECT: Contrast-enhanced computed tomography
mTOR: mammalian target of rapamycin
SAP-MS: Sodium acrylate and vinyl alcohol copolymer
TAE: Transcatheter arterial embolization
TSC: Tuberous sclerosis
